# Markers of inflammation and coagulation indicate a prothrombotic state in HIV-infected patients with long-term use of antiretroviral therapy with or without abacavir

**DOI:** 10.1186/1742-6405-7-9

**Published:** 2010-04-16

**Authors:** Eefje Jong, Joost CM Meijers, Eric CM van Gorp, C Arnold Spek, Jan W Mulder

**Affiliations:** 1 Department of Internal Medicine, Slotervaart Hospital, Amsterdam, the Netherlands; 2 Department of Infectious Diseases, University Medical Center, Utrecht, the Netherlands; 3 Department of Experimental Vascular Medicine, Academic Medical Center, Amsterdam, the Netherlands; 4 Department of Virology, Erasmus Medical Center Rotterdam, the Netherlands; 5 Center for Experimental and Molecular Medicine, Academic Medical Center, Amsterdam, the Netherlands

## Abstract

**Background:**

Abacavir (ABC) treatment has been associated with an increased incidence of myocardial infarction. The pathophysiological mechanism is unknown. In this study markers of inflammation and coagulation in HIV-infected patients using antiretroviral therapy with or without ABC were examined to pinpoint a pathogenic mechanism. Given the important role of high sensitivity C-reactive protein (hsCRP) levels in predicting cardiovascular risk, patient groups were also analyzed according to hsCRP levels.

**Methods:**

Patients treated with ABC and a matched control group treated without ABC were selected retrospectively. Vascular endothelial growth factor (VEGF) and markers of endothelial cell activation (von Willebrand factor (vWF), factor VIII), fibrin formation (fibrinogen, D-dimer, prothrombin fragment 1+2 (F1+2), endogenous thrombin potential (ETP)), anticoagulation markers (protein C and S, activated protein C sensitivity ratio (APCsr)) and inflammation markers (IL-6, hsCRP) were measured in citrated plasma.

**Results:**

A total of 81 patients were included of whom 27 patients used an ABC-containing regimen and 54 used a non-ABC-containing regimen. Patient characteristics were not significantly different between the groups except for longer duration of use of the current antiretroviral regimen in the ABC group (p = 0.01). The median time on ABC was 68 months (interquartile range 59-80 months). No differences in coagulation and inflammation markers according to ABC use were observed. For the whole patient group elevated vWF and F1+2 levels were observed in 23% and 37%, respectively. Compared to the reference ranges for the general population increased APCsr was found in 79% and lower protein C and VEGF levels in 40% and 43%, respectively. Patients in the high-risk category for cardiovascular disease with hsCRP levels > 3 mg/L had significantly higher fibrinogen, D-dimer, F1+2 and ETP levels compared to patients from the low-risk category with hsCRP levels < 1 mg/L.

**Conclusion:**

HIV-infected patients using ABC showed no specific abnormalities in coagulation or inflammation markers that might explain the increased risk of myocardial infarction. For the whole group, regardless of ABC use, evidence of a prothrombotic state was observed. Thirty-three percent of patients with long-term use of antiretroviral treatment had hsCRP levels above 3 mg/L, which is strongly associated with cardiovascular disease in HIV-uninfected individuals.

## Background

The Data Collection on Adverse Events of Anti-HIV Drugs (D:A:D) study, an observational study in over 30.000 HIV-1 infected individuals, reported an increased risk of myocardial infarction in HIV-infected patients with current or recent exposure to abacavir (ABC) and didanosine (ddI)[1]. The SMART study and a Danish study by Obel et al. observed a similar association with severe cardiovascular disease [2,3]. The increased risk was evident while patients were actually receiving the drugs up to 6 months after stopping them. However, the ACTG study group and GlaxoSmithKline-sponsored clinical trials observed no association between ABC use and increased risk of myocardial infarction or severe cardiovascular disease [4,5]. As possible pathogenic mechanisms endothelial dysfunction, a proinflammatory state with plaque rupture and subsequent thrombosis and platelet hyperreactivity have been suggested [6-9]. Earlier studies focussed on markers of inflammation and coagulation before and after initiation of an ABC-containing regimen. No changes in high sensitivity C-reactive protein (hsCRP), interleukin (IL)-6, soluble vascular adhesion molecule and D-dimer levels were observed [8,9]. In a recent study increases in metallopeptidase 9, myeloperoxidase and hsCRP levels as markers of cardiovascular risk were observed in a longitudinal cohort of virologically suppressed patients switching to ABC[10]. Earlier studies in non-HIV infected individuals described elevated high sensitivity C-reactive protein (hsCRP) levels as the most important factor in predicting cardiovascular risk [11]. Hsue et al. demonstrated lower flow-mediated vasodilatation as marker of endothelial dysfunction in patients on ABC [6]. Vascular endothelial growth factor (VEGF) could be an inducible factor in the process of endothelial dysfunction, but has not been studied in this setting. Anti-angiogenic properties through an inhibitory effect on VEGF were attributed to protease inhibitors in glioblastoma cells and treatment of Kaposi sarcoma [12,13]. Inhibition of VEGF was associated with thrombotic microangiopathy of the kidney [14].

In the current study markers of inflammation and coagulation in HIV-infected patients on present combined antiretroviral therapy (cART) with or without ABC were compared in order to pinpoint a pathogenic mechanism for the increased risk of myocardial infarction in patients using ABC. Given the important role of hsCRP levels in predicting cardiovascular risk, patient groups were also analyzed according to hsCRP levels.

## Methods

### Study population

All patients using an ABC-containing regimen were retrospectively selected from the Slotervaart HIV cohort study, a prospective cohort study on markers of coagulation and inflammation in HIV-infected patients. The control group existed of HIV-infected patients participating in the Slotervaart HIV cohort study who were using cART without ABC including a protease inhibitor (PI) or a non-nucleoside transcriptase inhibitor (NNRTI) and were matched for age, sex, CD4 cell count and HIV viral load. Patients using ddI were excluded. Blood was taken at a follow-up visit in the outpatient clinic, when the patient showed no signs of active infection.

### Laboratory testing

PT, aPTT and markers of endothelial cell activation (von Willebrand factor (vWF), VEGF)), fibrin formation (fibrinogen, D-dimer, prothrombin fragment 1+ 2 (F1+2), endogenous thrombin potential (ETP)), anticoagulation (protein C and S, activated protein C sensitivity ratio (APCsr)) and inflammation (IL-6, hsCRP) were measured in citrated plasma as described before[15] VEGF, hsCRP and IL-6 were assayed by ELISA (R&D Systems Europe Ltd., Abingdon, UK). The ETP assay is a global coagulation assay that measures thrombin generation in tissue factor triggered platelet-poor plasma, providing an estimation of the potential to form a clot under (patho)physiological conditions. The ETP was determined with a Calibrated Automated Thrombogram (CAT). The CAT assays the generation of thrombin in clotting plasma using a microtiter plate reading fluorometer (Fluoroskan Ascent, ThermoLab systems, Helsinki, Finland) and Thrombinoscope software (Thrombinoscope BV, Maastricht, the Netherlands) as previously described [16]. CD4 cell counts were analyzed using flow cytometric techniques (Becton Dickinson, USA). HIV RNA levels were quantified using the COBAS Ampliprep and COBAS TaqMan (Roche Diagnostics, Almere, The Netherlands).

### Statistical methods

Continuous variables were expressed as median values (interquartile range (IQR)) for not normally distributed variables and means (standard deviation) for normally distributed variables. Categorical variables were expressed as counts and percentages. Coagulation and inflammation markers were compared between patient groups with and without ABC. Besides, all patients were stratified in low-risk, average-risk and high-risk categories for cardiovascular disease according to their hsCRP levels independent of ABC use. hsCRP levels of <1.0 mg/L indicated low-risk, hsCRP levels of 1.0 - 3.0 mg/L indicated average-risk and hsCRP levels > 3.0 mg/L indicated high-risk for cardiovascular disease [11]. Normally distributed parameters were compared using a two-sample independent t-test. For not normally distributed parameters a Mann-Whitney test was used. Dichotomous variables were compared using a Chi-squared test. Categorical variables were analyzed using a Kruskall Wallis test with Bonferroni correction for multiple testing. A p-value of < .05 was considered significant. The calculations were performed using the Statistical Package for Social Sciences (SPSS Inc., Chicago, Illinois, version 16.0) software package.

## Results

A total of 81 patients were identified with a median age of 47 years (26-73 years), of whom 89% were male and 82% Caucasian. Twenty-seven patients were using an ABC-containing regimen and 54 a non-ABC-containing regimen. In both treatment groups 85% of patients were virologically suppressed for many years. One patient had a history of an ischemic cerebrovascular accident while on ABC before inclusion in the study. No patients with a documented myocardial infarction were reported. The patient characteristics stratified according to use of ABC are shown in Table 1. There were no significant differences between the groups except for longer duration of cART use in the patients treated with ABC (p = 0.01). The median time on ABC was 68 months (IQR 59-80 months).

**Table 1:** Patient characteristics according to ABC use

	ABC-containing regimen	Non-ABC-containing regimen
Number of patients (% male)	27 (89)	54 (89)
Age (years)	48 ± 11	47 ± 10
Ethnicity		
Caucasian (%)	23 (85)	43 (80)
African-American (%)	1 (4)	4 (7)
Asian (%)	2 (7)	4 (7)
Other (%)	1 (4)	3 (6)
Current smoker (%)	13 (48)	19 (35)
Chronic hepatitis C infection (%)	2 (7)	4 (7)
Chronic hepatitis B infection (%)	1 (4)	1 (2)
History of cardiovascular event (%)	1 (4)	-
Total duration of cART use (months)	116 (85-129)*	91 (33-121)
Total time on ABC (months)	68 (59-80)	-
cART regimen		
ABC + other NRTI (%)	11 (41)	-
ABC + PI (%)	7 (26)	-
ABC + NNRTI (%)	9 (33)	-
non-ABC + PI (%)	-	27 (50)
non-ABC + NNRTI (%)	-	27 (50)
CD4 cell count (cells/mm3)	490 (310-770)	530 (300-720)
HIV viral load <40 copies/ml (%)	23 (85)	46 (85)
Duration HIV viral load <40 copies/ml (months)	90 (72-113)	68 (22-120)
Total cholesterol (mmol/L)	5.0 ± 0.9	5.0 ± 1.0
Triglycerides (mmol/L)	2.5 ± 1.9	2.2 ± 1.7
Non-fasting glucose (mmol/L)	6.1 ± 1.2	5.9 ± 1.5
Use of antihypertensive drugs (%)	2 (7)	7 (13)
Use of statins (%)	7 (26)	6 (11)
Use of oral antidiabetics (%)	1 (4)	0 (-)

cART = combined antiretroviral therapy; ABC = abacavir; NRTI = nucleoside reverse transcriptase inhibitor; NNRTI = non-nucleoside reverse transcriptase inhibitor; PI = protease inhibitor

* p < 0.05

The median (IQR) of the laboratory markers according to ABC use are shown in Table 2. Table 3 shows the percentage of patients with a test result outside the reference range. No significant differences in laboratory parameters were observed between patients treated with and without ABC. However, for the whole group, we found elevated vWF levels in 23% of patients, elevated F1+2 levels in 37% of patients, while APCsr was increased in 79% of patients compared to the reference ranges for the general population. Low PC levels were observed in 40% and decreased VEGF levels in 43% of patients. IL-6 levels were low for the whole group. When stratified into risk categories for cardiovascular disease according to hsCRP levels, 28 (35%) patients fell in the low-risk category, 24 (30%) in the average-risk category and 27 (33%) in the high-risk category for cardiovascular disease. Patients on ABC were evenly distributed between the various categories. In Table 4 mean and median values for demographic and laboratory parameters are depicted grouped by risk category. Significantly higher fibrinogen, D-dimer, F1+2 and ETP levels were observed when the high-risk category was compared to the low-risk category. When only patients using ABC were selected this finding was confirmed for fibrinogen and D-dimer levels.

**Table 2:** Results of laboratory parameters according to ABC use*

Laboratory parameter	ABC-containing regimen (n = 27)	Non-ABC-containing regimen (n = 54)	Reference range for the general population
aPTT (sec)	30.8 (29.5-33.9)	31.4 (29.4-33.7)	25.0-38.0
PT (sec)	11.5 (11.0-12.0)	11.7 (11.1-12.2)	10.7-12.9
Fibrinogen (g/L)	2.9 (2.5-3.2)	2.7 (2.3-3.5)	1.9-4.0
FVIII (%)	119 (103-175)	119 (100-144)	63-173
vWF (%)	112 (89-185)	124 (100-168)	50-150
D-dimer (mg/L)	0.4 (0.2-0.4)	0.2 (0.2-0.4)	<1.00
F1+2 (pmol/L)	213 (177-276)	196 (122-257)	53-271
PC (%)	115 (98-138)	116 (98-127)	70-120
PS total (%)	105 (90-115)	104 (86-114)	58-130
PS free (%)	81 (68-102)	82 (70-99)	63-137
ETP (nM.min)	1844 (1620-2052)	1740 (1602-1959)	1155-2606
ETP peak (nM)	381 (345-394)	374 (339-414)	194-503
APCsr	2.2 (1.7-4.0)	2.5 (1.9-3.3)	<1.6
VEGF (pg/ml)	37 (27-47)	33 (23-51)	31-86
IL-6 (pg/ml)	<1.0 (-)	<1.0 (-)	<4.0
hsCRP (mg/L)	1.6 (0.4-5.3)	1.6 (0.5-3.3)	<1.0

*The results are shown as median values (interquartile range)

ABC = abacavir; fVIII = factor VIII; vWF = von Willebrand factor; F1+2 = prothrombin fragment 1+2; PC = protein C; PS = protein S; ETP = endogenous thrombin potential; APCsr = activated protein C sensitivity ratio; VEGF = vascular endothelial growth factor; hsCPR = high-sensitivity C-reactive protein

**Table 3:** Number (%) of patients with laboratory parameters outside the reference range

Laboratory parameter	Total group (n = 81)	ABC-containing regimen (n = 27)	Non-ABC-containing regimen (n = 54)
Prolonged aPTT	2 (3)	0 (-)	2 (4)
Prolonged PT	5 (6)	1 (4)	4 (7)
Elevated fibrinogen	11 (14)	3 (11)	6 (11)
Elevated FVIII	14 (17)	6 (22)	8 (15)
Elevated vWF	19 (23)	7 (26)	12 (22)
Elevated D-dimer	3 (4)	2 (7)	1 (2)
Elevated F1+2	30 (37)	10 (37)	20 (37)
Decreased PC	32 (40)	11 (41)	21 (39)
Decreased total protein S	6 (7)	3 (11)	3 (6)
Decreased free protein S	11 (14)	4 (15)	7 (13)
Increased ETP	0 (-)	0 (-)	0 (-)
Increased peak ETP	2 (3)	0 (-)	2 (4)
Increased APCsr	64 (79)	21 (78)	43 (80)
Decreased VEGF	4 (5)	2 (7)	2 (4)
Elevated IL-6	35 (43)	10 (37)	25 (46)
hsCRP category			
Low risk (hsCRP < 1 mg/L)	28 (35)	9 (33)	19 (35)
Average risk (hsCRP 1-3 mg/L)	24 (30)	6 (22)	18 (33)
High risk (hsCRP >3 mg/L)	27 (33)	10 (37)	17 (32)
Missing	2 (3)	2 (7)	-

ABC = abacavir; fVIII = factor VIII; vWF = von Willebrand factor; F1+2 = prothrombin fragment 1+2; PC = protein C; PS = protein S; ETP = endogenous thrombin potential; APCsr = activated protein C sensitivity ratio; VEGF = vascular endothelial growth factor; hsCRP = high-sensitivity C-reactive protein

**Table 4:** Demographic and laboratory parameters stratified to risk category based on hsCRP levels

	Low risk category (hsCRP < 1 mg/L)	Average risk category (hsCRP 1-3 mg/L)	High risk category (hsCRP >3 mg/L)
Number of patients	28	24	27
Age (years)	47.8 (± 10.2)	48.4 (± 11.6)	48.2 (± 9.1)
Duration of cART use (months)	76 (25-125)	116 (42-124)	111 (26-127)
CD4 cell count (cells/mm3)	390 (300-765)	460 (340-805)	500 (288-633)
HIV viral load <40 copies/ml (%)	24 (86)	21 (88)	22 (81)
Total cholesterol (mmol/L)	4.9 (± 1.0)	5.7 (± 0.9)	5.1 (± 0.8)
Triglycerides (mmol/L)	2.2 (± 1.9)	2.7 (± 2.0)	2.0 (± 1.4)
Non-fasting glucose (mmol/L)	5.9 (± 1.1)	5.9 (± 1.1)	6.1 (± 1.6)
Patients on ABC (%)	9 (32)	7 (29)	11 (41)
aPTT (sec)	31.5 (± 3.3)	32.2 (± 3.1)	30.5 (± 3.2)
PT (sec)	11.8 (± 0.9)	11.7 (± 0.6)	11.5 (± 0.8)
Fibrinogen (g/L)	2.4 (2.1-2.6)	2.8 (2.3-3.2)*	3.5 (2.8-4.1)*
FVIII (%)	115 (102-145)	122 (105-172)	122 (100-158)
vWF (%)	124 (92-160)	123 (101-177)	114 (98-188)
D-dimer (mg/L)	0.2 (0.2-0.3)	0.2 (0.2-0.3)	0.3 (0.2-0.5)*
F1+2 (pmol/L)	175 (151-229)	210 (163-269)	219 (197-305)*
PC (%)	114 (± 25.7)	111 (± 20.6)	120 (± 20.9)
PS total (%)	96 (± 18.7)	104 (± 14.5)	105 (± 20.8)
PS free (%)	87 (± 16.8)	93 (± 24.8)	82 (± 26.0)
ETP (nM.min)	1663 (± 274)	1803 (± 237)*	1913 (± 336)*
ETP peak (nM)	371 (328-401)	371 (341-407)	386 (364-417)
APCsr	2.9 (2.1-3.7)	2.0 (1.7-2.9)*	2.6 (1.6-4.4)
VEGF (pg/ml)	30 (24-42)	30 (26-52)	37 (27-53)
IL-6 (pg/ml)	1.0 (-)	1.0 (-)	1.0 (-)

hsCRP = high-sensitivity C-reactive protein; fVIII = factor; vWF = von Willebrand factor; F1+2 = prothrombin fragment 1+2; PC = protein C; PS = protein S; ETP = endogenous thrombin potential; APCsr = activated protein C sensitivity ratio; VEGF = vascular endothelial growth factor

*p < 0.05 (after Bonferroni correction for multiple testing)

## Discussion

We studied markers of inflammation and coagulation in HIV-infected patients treated with and without ABC to pinpoint a pathogenic mechanism for the increased risk of myocardial infarction in patients with current or recent (up to 6 months) exposure to ABC. Previous studies primarily focussed on markers of coagulation and inflammation before and after initiation of an ABC-containing regimen. In these studies changes in hsCRP, IL-6, soluble vascular adhesion molecule and D-dimer levels were not significantly different between patient groups treated with or without ABC [8,9]. We focussed on HIV-infected patients with long-term use of cART. Our findings showed no differences in inflammation and coagulation markers between HIV-infected patients treated with long-term cART with or without ABC.

In the current study, we hypothesized that VEGF, an important factor in the repair system of endothelial injury, might play a pathogenic role. VEGF is associated with angiogenesis, chemotaxis of macrophages and granulocytes, and vasodilatation. Anti-angiogenic properties through an inhibitory effect on VEGF were attributed to PIs in glioblastoma cells and treatment of Kaposi sarcoma [12,13]. No differences in VEGF levels were observed between patients on an ABC-containing regimen or a non-ABC-containing regimen. Nevertheless, VEGF levels were reduced in a significant proportion of the whole group of HIV-infected patients with long-term use of cART. This might be suggestive of a decrease in angiogenesis and endothelial repair. Use of PIs was evenly distributed between the two groups. No difference in VEGF levels was observed between patients using PIs or NNRTIs.

For the whole group evidence of endothelial cell activation, increased fibrin formation and decreased anticoagulation was observed compatible with a prothrombotic state. Furthermore, 33% of the patients with long term use of cART with undetectable or very low levels of viral replication had hsCRP levels >3 mg/L, which are strongly linked to cardiovascular disease in HIV-uninfected individuals [11]. IL-6 levels were low for the whole group indicating low levels of inflammation. A trend was observed of higher hsCRP levels in both the ABC- and non-ABC group with longer duration of cART use. Earlier Pallela et al. reported an increase in hsCRP levels between baseline and index visits (mean 4.2 years apart) in HIV-infected women on cART with or without ABC [9]. Higher hsCRP levels in HIV-infected individuals were also reported by Hsue et al. when comparing HIV-infected individuals to the general population [17]. In our study the high-risk category with hsCRP levels >3 mg/L showed increased fibrinogen, D-dimer, F1+2 and ETP levels indicating a prothrombotic state. No differences in markers of endothelial cell activation or anticoagulation according to hsCRP levels were observed.

The significant difference in duration of cART use could be a limitation of the study. If as hypothesized, ABC use would be associated with abnormal inflammation and coagulation markers this would probably be accentuated by longer duration of ABC use. Actually, no differences were observed between the two study groups. Furthermore, by using a cross-sectional design no conclusions could be drawn regarding the association of the observed inflammation and coagulation abnormalities and cardiovascular events.

## Conclusion

HIV-infected patients using ABC showed no specific abnormalities in coagulation or inflammation markers that might explain the increased risk of myocardial infarction. For the whole patient group, regardless of ABC use, evidence of a prothrombotic state was observed. Thirty-three percent of patients with long-term use of cART and undetectable viral load, had hsCRP levels above 3 mg/L, which is strongly associated with cardiovascular disease in HIV-uninfected individuals.

## Competing interests

EJ serves as a medical consultant to Gilead Medical Sciences.

## Authors' contributions

EJ, ECMG and JWM participated in the design of the study. JCMM carried out the coagulation assays. CAS carried out the hsCRP and IL-6 assays. EJ drafted the manuscript with input from the other authors. All authors read and approved the final manuscript.
